# Evaluating the validity of brief prototype‐based informant ratings of triarchic psychopathy traits in prisoners

**DOI:** 10.1002/bsl.2542

**Published:** 2021-10-17

**Authors:** Kelsey L. Lowman, Christopher J. Patrick, Emily R. Perkins, Gioia Bottesi, Maria Caruso, Paolo Giulini, Claudio Sica

**Affiliations:** ^1^ Department of Psychology Florida State University Tallahassee Florida USA; ^2^ Department of General Psychology University of Padova Padova Italy; ^3^ Department of Health Sciences Psychology Section University of Firenze Firenze Italy

**Keywords:** boldness, disinhibition, informant ratings, meanness, psychopathy, triarchic model

## Abstract

The validity of self‐report psychopathy assessment has been questioned, especially in forensic settings where clinical evaluations influence critical decision‐making (e.g., institutional placement, parole eligibility). Informant‐based assessment offers a potentially valuable supplement to self‐report but is challenging to acquire in under‐resourced forensic contexts. The current study evaluated, within an incarcerated sample (*n* = 322), the extent to which brief prototype‐based informant ratings of psychopathic traits as described by the triarchic model (boldness, meanness, disinhibition; Patrick et al., 2009) converge with self‐report trait scores and show incremental validity in predicting criterion measures. Self/informant convergence was robust for traits of boldness and disinhibition, but weaker for meanness. Informant‐rated traits showed incremental predictive validity over self‐report traits, both within and across assessment domains. These findings indicate that simple prototype‐based informant ratings of the triarchic traits can provide a useful supplement to self‐report in assessing psychopathy within forensic‐clinical settings.

## 1 INTRODUCTION

Researchers have long considered reliability and validity to be at the heart of sound measurement (Cronbach & Meehl, 1955), and contemporary experts in the clinical assessment field have continued to emphasize the importance of psychometric properties (Clark & Watson, 2019; Smith, 2005). In particular, multi‐source assessment has been endorsed as a promising means to enhance construct validity (Clark & Watson, 2019), and in a field dominated by self‐report, personality researchers have increasingly explored the corroborative and complementary benefits of informant‐rating data (Connelly & Ones, 2010). However, procedures for collecting informant data in research often involve detailed and time‐consuming protocols (i.e., semi‐structured interviews, multi‐item rating inventories) that do not extend well to forensic or correctional settings where resources of personnel, time, and money are limited. However, collateral sources of information are particularly important in settings of this type where assessment results can influence pivotal decisions such as institutional placement, enrollment in treatment, and eligibility for conditional release. For this reason, there is a critical need to evaluate the validity of briefer, more feasible methods for acquiring informant‐based assessment data in resource‐limited forensic and correctional settings.

### 1.1 Use of self‐report in assessing psychopathy

A prominent and clinically influential focus of evaluations in forensic‐correctional settings is on the assessment of psychopathic symptoms and propensities (Edens et al., 2018). Despite offering advantages such as efficiency, cost‐effectiveness, and access to subjective internal experiences, the utility of self‐report measures for assessing psychopathy has been questioned by prominent scholars in the field (for a review, see Sellbom et al., 2018). In particular, it has been argued that limitations inherent to self‐report, including susceptibility to response bias and deficient self‐insight, are of particular concern among psychopathic individuals and may render self‐report responses altogether untrustworthy in this group (Hart et al., 1994; Lilienfeld, 1994; Sellbom et al., 2018). These arguments are grounded in classic depictions of psychopathy that underscore pathological deceitfulness at the core of the disorder, along with a lack of self‐awareness or insight (e.g., Cleckley, 1976).

Concerns about the validity of self‐report based measures have been raised especially in forensic and legal contexts, where psychopathy assessments frequently factor into critical decisions such as sentencing or eligibility for parole (Edens et al., 2001, 2018; Skeem et al., 2011). Skeptics contend that psychopathic individuals facing these decisions have both the incentive and capacity to distort their responses to work in their favor, based on whether they stand to benefit from forging a positive impression (i.e., parole eligibility) or a negative one (i.e., malingering; Edens et al., 2000; Rogers & Cruise, 2000). Research on these assertions, however, suggests that while psychopathic individuals may endorse greater aptitude for and willingness to engage in malingering (Edens et al., 2000), their efforts to do so are not necessarily successful (Edens et al., 2000; Poythress et al., 2001). Moreover, a growing body of literature suggests that psychopathic individuals are generally able (Miller et al., 2011) and willing (Ray et al., 2013; Watts et al., 2016) to provide accurate self‐report. It should be cautioned, however, that such findings are limited to confidential research settings where honest reporting does not risk forensic or legal repercussions.

### 1.2 Use of informant report in assessing psychopathy

As a complement to any questionable reliability associated with self‐report, informant report offers a pragmatic alternative or supplement for assessing psychopathy and personality pathology more broadly (Miller & Lynam, 2015; Vazire, 2006). On one hand, correspondence between self and informant report may depend in part on the extent of familiarity within rating pairs, especially for evaluating less conspicuous traits (i.e., affective experience) compared to more observable ones (i.e., interpersonal and behavioral dispositions) (Connelly & Ones, 2010). At the same time, any unique information provided by informant data may buffer inaccuracies that conceivably undermine self‐report (whether introduced by response bias or lack of self‐awareness), thus improving the reliability and validity of personality assessment overall (Connolly et al., 2007; Klonsky et al., 2002; Markon et al., 2013; Miller & Lynam, 2015). In particular, informant ratings may be useful for evaluating ego‐dystonic features, or those that conflict with the ideal self‐image, that could be more vulnerable to self‐distortion or positive impression management (i.e., manipulativeness, proactive aggression) (Sellbom et al., 2018; Vazire, 2010). Moreover, Meehl (1986) advocated for pursuing an “open” approach to construct definition, such that all available sources of information are evaluated as potential indicators of the latent construct to maximize content coverage. In the realm of psychopathy, construct validity thus stands to benefit from considering collateral data, especially to the extent it provides incremental information over and above self‐report data with respect to conceptualizing psychopathy and meaningfully extending its nomological network (Cronbach & Meehl, 1955; Lilienfeld, 1994; Meehl, 1959, 1986).

### 1.3 Self/informant convergence

To date, a limited number of studies have investigated self/informant convergence in psychopathy assessment, with the magnitude of reported correlations generally ranging from modest to moderate (*r*s ≈ 0.23–0.62; e.g., Fowler & Lilienfeld, 2007; Gerbrandij et al., 2019; Iyican et al., 2015; Kelley et al., 2018). Notably, these studies span a range of samples with varying degrees of rater/participant familiarity, including undergraduates (Fowler & Lilienfeld, 2007; Kelley et al., 2018), couples reporting intimate‐partner violence (Iyican et al., 2015), and forensic patients (Gerbrandij et al., 2019). In addition to showing covariance between informant ratings and self‐report, several studies have demonstrated that informant ratings of psychopathy predict clinical criterion variables such as antisocial behavior (Gerbrandij et al., 2019; Jones & Miller, 2012; Kelley et al., 2018).

However, predictors generally exhibit stronger associations with criterion measures assessed in the same measurement modality, owing to shared method variance—that is, covariance attributable to methodological similarities (Blonigen et al., 2010; Campbell & Fiske, 1959). For example, in a study of couples who endorsed male‐perpetrated intimate partner violence (IPV; Iyican et al., 2015), males' self‐reported psychopathy predicted their self‐report of IPV engagement, but not their female partner's collateral report of male‐perpetrated IPV. In turn, females' informant report of male psychopathy predicted their collateral report of male‐perpetrated IPV, but not males' self‐report of inflicted IPV. Similarly, Gerbrandij et al. (2019) found that validity coefficients for self‐report psychopathy scores were generally stronger for self‐report criterion variables (e.g., psychopathology symptoms) compared to informant‐rated criterion variables (e.g., risk‐assessment estimates), whereas the opposite was true for informant‐rated psychopathy scores. These findings underscore the moderating effect of method variance on the predictive validity of assessments, and are in line with research by Blonigen et al. (2010) demonstrating weaker relations between psychopathy scores and criterion measures across modes of assessment (e.g., interview and self‐report) as compared to within‐mode (i.e., interview only).

The wide variety of psychopathy inventories employed by different research groups is another factor that impedes the synthesis and interpretation of findings across studies of self‐ and other‐reported psychopathic traits. For example, Iyican et al. (2015) collected self and partner reports on a 56‐item short form of the Psychopathic Personality Inventory (PPI‐SF; Lilienfeld & Hess, 2001), which yields scores on eight content scales and two higher‐order factor scores (Fearless Dominance, Impulsive Antisociality). Another study by Fowler and Lilienfeld (2007) computed a global psychopathy score from scores on the Psychopathy Q‐Sort (Reise & Oliver, 1994) administered separately to participants and peer informants. Both studies reported significant self/other convergence (*r*s = 0.31–0.62 for Iyican et al., 2015; *r* = 0.32 for Fowler & Lilienfeld, 2007), but it is difficult to meaningfully juxtapose the two sets of findings when psychopathy is treated as a multifaceted construct in one study and as unitary in the other. Such disagreement surrounding the definition and structure of psychopathy has pervaded the field of psychopathy research since its origin (e.g., Lilienfeld et al., 2016; Skeem & Cooke, 2010), and may continue to hinder the generalizability of research findings until consensus is reached in these respects.

### 1.4 Triarchic model of psychopathy

The triarchic model was introduced to reconcile conflicting theories of psychopathy and serve as a basis for integrating findings from studies employing different conceptual frameworks and assessment methods (Patrick et al., 2009). According to this model, psychopathy encompasses three distinct characteristics—boldness, meanness, and disinhibition—each of which is conceptualized in trait‐dispositional and biobehavioral terms. Boldness entails interpersonal dominance, emotional resiliency, and fearlessness, and is associated with reduced physiological sensitivity to threat (Esteller et al., 2016; Yancey et al., 2019) and low susceptibility to internalizing problems (Latzman et al., 2020). Meanness embodies features of callousness, selfishness, and antagonistic victimization and is theorized to reflect low affiliative capacity (Palumbo et al., 2020; Viding & McCrory, 2019). Disinhibition involves general proneness to externalizing problems and has been linked to weaknesses in neural systems for inhibitory control and executive functioning (Venables et al., 2018). Support for the triarchic model as an integrative framework comes from writings showing how the triarchic traits are represented in different historic accounts of psychopathy (Crego & Widiger, 2016; Patrick et al., 2009) and studies demonstrating representation of these traits in various inventories for assessing it (Drislane et al., 2014; Patrick & Drislane, 2015).

To date, two studies have examined the convergence and comparative predictive validity of self‐ and other‐reported psychopathic traits as described by the triarchic model (Gerbrandij et al., 2019; Kelley et al., 2018). In a sample of undergraduate roommate pairs, Kelley et al. (2018) evaluated convergence between self‐reported and informant‐rated traits, assessed in each modality using items from the Triarchic Psychopathy Measure (Patrick, 2010), and compared the two as predictors of antisocial behavior also assessed in each modality. Informant‐rated traits were moderately correlated with their self‐report counterparts (*r*s = 0.36–0.54). Informant ratings of all three triarchic traits showed higher validity coefficients for informant‐assessed criterion variables, and informant report did not contribute incrementally to the prediction of any self‐report criteria over/above self‐report triarchic scores. In another separate study, Gerbrandij et al. (2019) computed scores for the triarchic traits using content‐relevant items from self‐report and informant‐rating versions of the Schedule for Nonadaptive and Adaptive Personality—Forensic Version (SNAP‐F; Keulen‐de‐Vos et al., 2011) administered, respectively, to a sample of forensic psychiatric inpatients and clinical staff personnel who knew them. Informant ratings of the triarchic traits in this study correlated modestly to moderately with self‐report trait scores (*r*s = 0.23–0.45) and predicted several criterion variables in both domains, with disinhibition ratings contributing to prediction of self‐reported symptoms in areas of anxiety, depression, hostility, and paranoid ideation (Gerbrandij et al., 2019). Once again, however, informant ratings demonstrated greater predictive validity for criterion variables within the same assessment modality and did not exhibit incremental validity over/above self‐reported traits in predicting self‐report criterion variables. Importantly, both of these studies had informants use an item‐based rating measure that was as detailed as the self‐report inventory completed by participants, raising the question of whether a more streamlined, time‐efficient informant rating method might exhibit higher convergence with self‐report and show greater evidence of incremental validity.

### 1.5 Current study

In correctional settings where the validity of self‐report assessed psychopathy is of particular concern (e.g., Rogers & Cruise, 2000), it is critical to investigate the practicality and comparative validity of informant‐rating methods of assessment. To the extent that self and informant ratings of psychopathy overlap or provide unique information about clinically important outcomes, informant data may serve as a valuable tool to improve the validity of psychopathy assessment. In the context of making crucial decisions that frequently take personality features into account (i.e., granting occasional release or formulating a reintegration prognosis), accurate appraisal of psychopathic features among incarcerated individuals is essential.

The current study investigated the degree to which informant ratings of boldness, meanness, and disinhibition converge with self‐reports of the same traits, and compared the predictive validity of the two in relation to various criterion measures, in a sample of incarcerated individuals. Given the prevalence of barriers like overcrowding and limited resources in correctional settings, informant data in this study were collected using a brief, easily implemented rating procedure based on prototypic descriptions of boldness, meanness, and disinhibition. We hypothesized that (1) convergence between informant ratings and counterpart self‐report scores would be significant, but likely lower than those reported by Kelley et al. (2018) and Gerbrandij et al. (2019) given our use of a simplified rating method that differs from its self‐report counterpart in both information source (self vs. informant) and report format (item‐based vs. global rating). However, we expected that (2) informant trait ratings would still show evidence of predictive validity in relation to criterion measures. Finally, we evaluated (3) whether informant ratings showed incremental validity in predicting criterion measures over and above self‐report scores.

## 2 METHODS

### 2.1 Participants

The study sample (*n* = 362) consisted of 277 incarcerated males and 85 incarcerated females from 10 separate medium‐ and high‐security prisons in Italy. The National Administration of Prisons (*Dipartimento dell'Amministrazione Penitenziara*; DAP) and its local division for the Lombardy region in Northern Italy (PRAP Regione Lombardia) granted approval for data collection at these 10 prisons. Study procedures adhered to the Declaration of Helsinki and were approved by the DAP and by the Institutional Review Board of the University of Firenze.

Study personnel reviewed prison files to determine eligibility in relation to exclusion criteria as follows: (i) criminal history limited to minor property offenses or illicit substance use/trafficking in small quantities (ii) current serious mental illness (i.e., schizophrenia, Bipolar I disorder), or intellectual disability, (iii) involvement with the institution's Drug Addiction Services over the last six months (i.e., entailing pharmacological treatment such as methadone administration to mitigate withdrawal and craving symptoms from severe substance use disorder), (iv) lack of fluency in the Italian language, (v) visual or hearing impairments, and (vi) impending release from the prison. Eligible individuals were invited by case managers at each prison to participate on a completely voluntary basis. No direct incentives were offered for participation in the study, and there were no negative consequences for declining to participate. For those eligible and invited to participate, the enrollment rate was approximately 95%, with no substantial differences across the 10 prison facilities.

A total of 49 case managers across the 10 prisons volunteered to serve as informant raters in the study. Each case manager rated between 3 and 16 prisoners (mean = 6, SD = 3.5) so that each prisoner was rated once. Case managers were given prototypic descriptions of boldness, meanness, and disinhibition (see Section 2.2 below for further details, and Supplemental Material for copies of descriptions) and were asked to provide three separate ratings for each prisoner indicating the degree to which they exemplified each trait. Additionally, case managers provided ratings for each participant's behavior in prison, social connections outside of prison, and prognosis for reintegration into society (see Section 2.2 for details). In Italy, case managers typically have background training in social work or professional education. However, the DAP prohibited obtaining any information about individual case managers for privacy and security reasons. As a result, data are not available regarding each case manager's length of employment at each prison or degree of familiarity with each participant they rated (see Section 4.5 of the Discussion for further consideration of this issue).

Case managers serving as informants first provided their ratings for eligible incarcerated individuals at the start of data collection. Next, incarcerated individuals who elected to participate provided informed written consent prior to participation in the study. In small groups, enrolled participants then completed demographic items (age, education, and marital status), followed by the self‐report measures described in the Section 2.2 below, which were administered in a counterbalanced manner to control for order effects.

Four male and two female participants provided incomplete self‐report data and were omitted from analyses. 10 participants for whom informant ratings were not provided by case managers were also omitted. Multivariate outliers were excluded following the procedure described by Leys and colleagues, in which a variant of the Mahalanobis distance based on the Minimum Covariance Determinant was used (Leys et al., 2018), employing a breakdown point of 0.25 (i.e., assuming less than 25% outliers in our sample) and a detection level of *p* < 0.01. This resulted in 24 participants (7% of the sample with full questionnaire data) being excluded, yielding a final analysis sample of 322.

The mean age of the final analysis sample was 45.9 years (SD = 12.6 years, range 20‐72), with an average of 9.4 years of education (SD = 3.5 years). Approximately one‐third of the sample was single (34.5%), 37.3% were married or cohabitating, 21% were separated or divorced, and 7% were widowed. Additional demographic data such as race, ethnicity, and gender identity were not collected. Nearly all participants had a history of multiple criminal offenses; however, for a large portion of the sample, data were not available to characterize the nature of prior convictions. Prison personnel reported the offense of the most serious type associated with each participant's current sentence: 21% were convicted of homicide, 20% pedophilia/sexual aggression, 17% armed robbery, 10% fraud and extortion, 10% Mafia affiliation, 7% drug trafficking, 7% aggression and fighting, 6% criminal conspiracy, and 3% bankruptcy. About 8% (*n* = 25) of the participants in the final analysis sample were serving life sentences. The remaining 297 were serving an average sentence of 9.7 years (SD *=* 8.0 years).

### 2.2 Measures

#### 2.2.1 Psychopathy measures

##### Self‐report: Triarchic Psychopathy Measure (TriPM)

The 58‐item TriPM (Patrick, 2010) provides scores on three subscales—Boldness, Meanness, and Disinhibition—representing the traits of the triarchic model of psychopathy (Patrick et al., 2009). Participants respond to each item on a 4‐point Likert scale where 0 = *false*, 1 = *somewhat false*, 2 = *somewhat true*, and 3 = *true*. The 19‐item Boldness subscale indexes the general factor of a multi‐scale inventory of boldness (Patrick et al., 2019) through items measuring social dominance, resilience to stress, and fearlessness. The TriPM Disinhibition scale is composed of 20 items from the Externalizing Spectrum Inventory (ESI; Krueger et al., 2007) that index its general externalizing factor, and the TriPM Meanness scale consists of 19 ESI items designed to index its callous‐aggression subfactor. The Italian version of the TriPM (Sica et al., 2015) used in this study has shown good psychometric properties in other research (e.g., Brislin et al., 2019; Sica et al., 2015; Somma et al., 2019). Within the current sample, the TriPM scales exhibited satisfactory internal consistencies (Cronbach's alpha) and mean corrected item‐total correlations (*r*): αs for Boldness, Meanness, and Disinhibition = 0.70, 0.87, and 0.85, respectively, *M* item‐total *r*s = 0.28, 0.47, and 0.42.

##### Informant report: triarchic prototype ratings

Informant ratings were based on prototypic descriptions of boldness, meanness, and disinhibition generated by Hall et al. (2014) and used by these and subsequent authors (e.g., Brislin et al., 2015; Drislane et al., 2015) as referents for developing new triarchic scale sets. For the current study, we shortened and simplified these descriptions, and translated them from English to Italian using a recursive process (cf. Brislin, 1986) of translation, back‐translation, and review by an expert on the triarchic model constructs. The prototypic descriptions used in this study are provided in both English and Italian versions in the online Supplemental Material. Informants read the prototypic descriptions of the three triarchic traits and rated designated prisoner participants on each using a Likert scale from 1 (*does not describe this person at all*) to 7 (*perfectly describes this person*).

#### 2.2.2 Criterion measures

##### Self‐report measures

Self‐report criterion measures for the current study consisted of inventories assessing depressive hopelessness, propensities toward self‐harm, and substance problems. The first of these, the Beck Hopelessness Scale (BHS; Beck et al., 1974), comprises 20 true‐false items covering feelings about the future, loss of motivation, and negative expectations. Participants endorse an item as true if it adequately describes their emotional state over the past week. Extensive research has supported the BHS as predictive of self‐harm and suicidal behavior (McMillan et al., 2007), and the Italian version (Pompili et al., 2009) used in this study has demonstrated good reliability and validity in other research (Pompili et al., 2007). In the current sample, Cronbach's alpha (*α*) was 0.86 and the mean‐corrected item‐total correlation (*r*) was 0.52.

The self‐harm measure was the Deliberate Self‐Harm Inventory (DSHI; Gratz, 2001), a 17‐item lifetime assessment of voluntary self‐inflicted injury performed without suicidal intent, but serious enough to damage bodily tissue. A lifetime frequency score is derived from the participant's total lifetime number of self‐harm episodes. These episodes are further broken down into self‐harm modality (e.g., cutting, burning, head banging, etc.), yielding a versatility score that represents the number of different modalities of self‐harm experienced. Self‐harm frequency scores were log‐transformed to normalize their distribution, and these transformed scores correlated highly with versatility scores (*r* = 0.82). The Italian translation used in the current study has shown good reliability in other research (Cerutti et al., 2012), and Cronbach's alpha for the versatility score was acceptable in the current sample (*α* = 0.70).

The Substance Abuse factor scale from the ESI (ESI‐Sub; Patrick et al., 2013) includes nine items that index problematic use of alcohol, marijuana, and other substances. Items of this scale are rated on a 4‐point Likert scale where 0 = *false*, 1 = *somewhat false*, 2 = *somewhat true*, and 3 = *true*. One third of the items assess alcohol‐specific problems (e.g., “I've often ended up drinking more than I should”), one third relate to marijuana (e.g., “I've had urges to use marijuana that were hard to resist”), and the final third reference other substances (e.g., “I've used downers like Valium or Xanax for non‐medical reasons”). For the substance use problem scale in the current sample, Cronbach's α was 0.90 and the mean‐corrected item‐total *r* was 0.55.

##### Informant (case manager) ratings

As mentioned above, case managers rated each prisoner on three aspects of adjustment, using one to five scales for which higher values represented more favorable ratings: (i) behavior in prison (1 = *very bad* to 5 = *very good*), (ii) social connectedness with relatives or friends outside prison (1 = *none, isolated* to 5 = *important and stable social links outside prison*), and (iii) reintegration prognosis (1 = *negative, many failures in the past* to 5 = *positive and favorable*). Case managers made these ratings based on information contained in prison files together with observations of or direct experiences with the designated prisoner in various contexts (e.g., case‐related meetings, supervised release outings [e.g., funeral attendance]). Archival information in prison files included records of social contacts (phone calls, visitors, etc.) and reports concerning daily behavior filed by correctional staff.

### 2.3 Data analysis

Skewness and kurtosis were evaluated for all variables, and self‐harm frequency scores were log‐transformed (as noted) to better approximate normality. In the case of missing score values, expectation‐maximization (EM) algorithm‐based maximum likelihood estimates were computed and utilized in analyses. Simple regression analyses were used to quantify how informant ratings for Boldness, Meanness, and Disinhibition predicted self‐report trait scores and other criterion variables, both individually and alongside the other two traits, when accounting for variation in scores due to differing raters. To account for rater effects, raters were assigned numbers and represented in regression analyses through the use of dummy‐coding. Hierarchical regression analyses were performed to evaluate whether informant ratings of traits contributed incrementally to prediction of criterion measures over and above self‐report trait scores. In these analyses, dummy‐coded variables representing different raters were entered in Step 1, self‐reported Boldness, Meanness, and Disinhibition were entered in Step 2, and informant‐rated Boldness, Meanness, and Disinhibition were entered in Step 3.

## 3 RESULTS

Table 1 presents means and standard deviations for self‐report and informant‐rated Boldness, Meanness, and Disinhibition. To facilitate comparisons between the two report modalities, Table 1 also reports each scale mean as a percentage of the maximum possible score (POMP; Cohen et al., 1999) for that scale (i.e., percentage of 57‐60 for TriPM scales, 7 for informant ratings). For self‐report scores, Boldness correlated modestly with Meanness (*r* = 0.16, *p* < 0.01) but was not associated with Disinhibition (*r* = −0.02, *p* = 0.67), and Meanness correlated moderately with Disinhibition (*r* = 0.55, *p* < 0.001). Comparatively, inter‐correlations among informant‐rated traits were stronger: Boldness correlated modestly with both Meanness (*r* = 0.31, *p* < 0.001) and Disinhibition (*r* = 0.26, *p* < 0.001), and Meanness and Disinhibition ratings were strongly correlated (*r* = 0.65, *p* < 0.001).

**TABLE 1:** Means (*M*), standard deviations (SD), and percentage of maximum possible score (POMP; Cohen et al., 1999) for self‐report and informant‐rating scores on boldness, meanness, and disinhibition in the full study sample (*n* = 322)

	*M*	SD	POMP
Self‐report (TriPM)			
Boldness	27.24	7.74	47.79
Meanness	12.00	8.55	21.05
Disinhibition	21.79	11.28	36.32
Informant‐rating			
Boldness	3.32	1.75	38.67
Meanness	2.12	1.34	18.67
Disinhibition	2.69	1.63	28.17

*Note*: Range of possible scores for TriPM Boldness and Meanness is 0–57, and for TriPM Disinhibition, 0–60. Range of possible scores for each informant‐rating variable is 1–7.

Abbreviation: TriPM, Triarchic Psychopathy Measure.

Table 2 presents standardized beta coefficients for informant‐rated triarchic traits with (i) self‐report (TriPM) trait scores, (ii) self‐report criterion variables (hopelessness, self‐harm frequency/versatility, and substance use problems), (iii) informant‐rated criterion variables (case manager ratings for behavior in prison, social connectedness, and reintegration prognosis), and (iv) years of sentence. For each informant‐rated triarchic trait, Table 2 shows standardized beta coefficients (*β*s) from (a) regression analyses including only that trait along with dummy‐coded raters as predictors (left columns, labeled *β*_B_, *β*_M_, and *β*_D_, respectively), and (b) regression analyses including that trait together with the other two, along with dummy‐coded raters, as predictors (right columns, labeled *β*_T_ in each case). Correlations between self‐report triarchic trait scores and criterion variables in this sample were reported previously by Brislin et al. (2019) and thus are not presented in the current paper.^1^

**TABLE 2:** Standardized regression coefficients for informant‐rated triarchic trait scores when entered separately (*β*_B_, *β*_M_, *β*_D_), and all together (*β*_T_), as predictors of self‐report based triarchic (TriPM) scores and different‐modality criterion variables, after controlling for rater effects

Criterion measure	Informant‐rated trait						*R* (*R* ^2^)^a^
	Boldness		Meanness		Disinhibition		
	*β*_B_	*β*_T_	*β*_M_	*β*_T_	*β*_D_	*β*_T_	
Self‐report criteria							
TriPM boldness	0.26***	0.24***	0.12	−0.01	0.15*	0.08	0.23 (0.05)
TriPM meanness	0.02	−0.03	0.18*	0.14	0.15*	0.07	0.15 (0.02)^b^
TriPM disinhibition	0.02	−0.06	0.20**	0.02	0.30***	0.31***	0.24 (0.06)
Hopelessness	−0.25**	−0.28**	0.04	0.13	0.01	−0.01	0.24 (0.06)
Self‐harm frequency	−0.02	−0.08	0.13	0.00	0.21**	0.24**	0.18 (0.03)
Self‐harm versatility	−0.05	−0.11	0.10	−0.04	0.19**	0.25**	0.18 (0.03)
Substance use problems	0.16**	0.06	0.34***	0.17*	0.36***	0.23**	0.31 (0.09)
Informant‐rated criteria							
Behavior in prison	−0.03	0.11*	−0.39***	−0.13	−0.49***	−0.43***	0.40 (0.16)
Social connectivity	0.11	0.21***	−0.26***	−0.17	−0.28***	−0.24**	0.30 (0.09)
Reintegration prognosis	−0.02	0.14*	−0.47***	−0.22**	−0.52***	−0.42***	0.45 (0.20)
Years of sentence	0.19**	0.18**	0.12	0.13	0.04	−0.10	0.18 (0.03)

*Note*: All analyses used dummy coding to control for rater effects. Left column for each informant‐rated triarchic trait lists beta coefficients from regression models including only that trait along with dummy‐coded raters as predictors (*β*_B_ = including Boldness only; *β*_M_ = including Meanness only; *β*_D_ = including Disinhibition only); right column for each trait lists beta coefficients from regression models including that trait together with the other two, along with dummy‐coded raters, as predictors (*β*_T_ = including all three triarchic traits).

Abbreviation: TriPM, Triarchic Psychopathy Measure.

^a^ *R* and *R* ^2^ values reflect variance explained by the three informant ratings together, in the joint regression model.

^b^ Omnibus *R*s were significant (*p* < 0.05) for all prediction models except this one.

*p < 0.05, **p < 0.01, ***p < 0.001.

### 3.1 Self/informant agreement in assessing triarchic traits

In both individual‐trait and joint‐trait regression analyses, informant‐rated Boldness was preferentially related to its self‐report counterpart (*β*_B_ = 0.26, *β*_T_ = 0.24, *p*s < 0.001). As shown in Table 2, informant‐rated Disinhibition predicted self‐reported Boldness on its own (*β*_D_ = 0.15, *p* < 0.05), but not in the regression model including all three ratings as predictors (*β*_T_ = 0.08, *p* = 0.35). Informant‐rated Meanness did not predict self‐reported Boldness. Meanness and Disinhibition ratings each significantly predicted self‐reported Meanness when evaluated individually (*β*_M_ = 0.18 and *β*_D_ = 0.15, *p*s < 0.05), but when included together along with boldness in the joint regression model, the beta coefficient for informant‐rated Meanness fell short of significance (*β*_T_ = 0.14, *p* = 0.15) and the coefficient for Disinhibition was clearly nonsignificant (*β*_T_ = 0.07, *p* = 0.43)—indicating that variance shared between the two traits contributed to the independent beta coefficients for each. Significant beta coefficients were also evident for both informant‐rated Meanness (*β*_M_ = 0.20, *p* < 0.01) and Disinhibition (*β*_D_ = 0.30, *p* < 0.001) when evaluated individually as predictors of self‐reported Disinhibition, but the joint regression model in this case revealed a unique predictive association for informant‐rated Disinhibition (*β*_T_ = 0.31 *p < *0.001) but not for Meanness (see Table 2).

### 3.2 Predictive validity of informant ratings

#### 3.2.1 Self‐report criterion variables

Hopelessness was associated with lower informant‐rated Boldness in both the individual‐trait and joint‐trait regression models (*β*_B_ = −0.25, *β*_T_ = −0.28, *p*s < 0.001), whereas it was unrelated to informant‐rated Meanness or Disinhibition (see Table 2). Self‐harm frequency and versatility, on the other hand, were related positively to informant‐rated Disinhibition (*β*_D_ = 0.21 and 0.19, *β*_T_ = 0.24 and 0.25, respectively, *p*s *<* 0.01), but not to either Boldness or Meanness. Substance use problems showed positive associations with all three informant‐rated traits when evaluated individually (*β*_B_ = 0.16, *p* < 0.01; *β*_M_ = 0.34, *β*_D_ = 0.36, *p*s < 0.001), though only Meanness and Disinhibition ratings significantly predicted substance use problems in the joint regression model (*β*_T_ = 0.17, *p* < 0.05 and *β*_T_ = 0.23, *p < *0.01, respectively).

#### 3.2.2 Informant‐rated criterion variables

In individual‐trait regression models, ratings for behavior in prison were negatively associated with both Meanness and Disinhibition (*β*_M_ = −0.39 and *β*_D_ = −0.49, *p*s < 0.001), but not Boldness. However, when the three informant‐rated traits were entered together in a regression model, informant‐rated Boldness positively predicted prison behavior ratings (*β*_T_ = 0.11, *p < *0.05), whereas Disinhibition predicted worse behavior ratings (*β*_T_ = −0.43, *p < *0.001) and Meanness was no longer significant. The same pattern was evident for the criterion of informant‐rated social connectivity: Meanness and Disinhibition but not Boldness emerged as significant in their individual regression models (*β*_M_ = −0.26 and *β*_D_ = −0.28, *p*s < 0.001), but in a joint regression model with all three traits predicting social connectivity, Boldness predicted better ratings (*β*_T_ = 0.21, *p < *0.001), Disinhibition predicted worse ratings (*β* = −0.24, *p < *0.01), and Meanness was no longer significant. Similarly, for informant‐rated reintegration prognosis, the individual regression analyses yielded significant predictive effects for informant‐rated Meanness and Disinhibition (*β*_M_ = −0.47 and *β*_D_ = −0.52, *p*s < 0.001), but all three traits emerged as significant predictors in the joint regression analysis. Again, Boldness predicted more favorable reintegration prognosis ratings (*β*_T_ = 0.14, *p < *0.05), whereas Meanness (*β*_T_ = −0.22, *p < *0.01) and Disinhibition (*β*_T_ = −0.42, *p < *0.001) were associated with poorer ratings.

#### 3.2.3 Years of sentence

Across all regression analyses, years of sentence was significantly predicted only by informant‐rated Boldness (*β*_B_ = 0.19, *β*_T_ = 0.18, *p*s *<* 0.01). Informant‐rated Meanness and Disinhibition did not significantly predict years of sentence either on their own, or alongside the other informant‐rated traits.

### 3.3 Incremental validity of informant ratings

Table 3 presents standardized beta coefficients from Step 3 of hierarchical regression analyses (at which point informant‐rated Boldness, Meanness, and Disinhibition were added as predictors of each criterion variable, following inclusion of self‐report scores for these traits at Step 2 and dummy‐coded raters at Step 1). Rater effects in the current study were large (range of *R* ^2^ values at Step 1 = 0.19–0.34). To avoid misrepresenting the degree of variance in each criterion measure explained by self‐ and informant‐report, Table 3 presents only the Δ*R* ^2^ values for Step 2 (reflecting the incremental contribution of self‐report scores over and above rater effects) and Step 3 (reflecting the incremental contribution of informant ratings over and above self‐report scores and rater effects). Based on significant Δ*R* ^2^ values, informant‐rated triarchic traits contributed incrementally to the prediction of self‐reported hopelessness and substance use problems, all informant‐rated criterion variables, and years of sentence. For these models in which significant contributions were evident, the mean increase in variance explained by adding the informant‐rating scores at Step 3 was 7.2%.

**TABLE 3:** Results from hierarchical regression models predicting criterion variables from self‐report triarchic scores (Step 2) and informant‐rated triarchic scores (Step 3), after controlling for rater effects at Step 1

Criterion measure	Self‐report			Informant report			Model	
	Bold	Mean	Dis	Bold	Mean	Dis	Step 2:	Step 3:
	*β*	*β*	*β*	*β*	*β*	*β*	Δ*R* ^2^	Δ*R* ^2^
Self‐report criteria								
Hopelessness	−0.30***	0.23***	0.09*	−0.19**	0.10	−0.03	0.15***	0.03*
Self‐harm frequency	−0.12*	0.11	0.23**	−0.04	−0.02	0.17	0.10***	0.01
Self‐harm versatility	−0.06	0.03	0.23**	−0.08	−0.05	0.18*	0.07***	0.01
Substance use problems	0.04	−0.03	0.49***	0.08	0.17*	0.07	0.22***	0.04***
Informant‐rated criteria								
Behavior in prison	−0.11*	−0.06	−0.17**	0.13*	−0.12	−0.37***	0.09***	0.11***
Social connectivity	0.03	−0.06	0.00	0.20**	−0.16	−0.24**	0.01	0.08***
Reintegration prognosis	−0.09	0.06	−0.21**	0.15*	−0.23**	−0.34***	0.08***	0.15***
Years of sentence	−0.04	−0.03	0.05	0.19**	0.14	−0.11	0.00	0.03**

*Note*: All analyses used dummy coding to control for rater effects. Dummy‐coded variables representing different raters were entered in Step 1, self‐reported TriPM Boldness, Meanness, and Disinhibition scores were entered in Step 2, and informant‐rated Boldness, Meanness, and Disinhibition scores were entered in Step 3. Standardized beta values (*β*) listed are for Step 3 of the model. Step 2 Δ*R* ^2^ = change in proportion of total variance accounted for in each criterion variable from Step 1 to Step 2 (i.e., after adding self‐report TriPM scores); Step 3 Δ*R* ^2^ = change in proportion of total variance accounted for in each criterion variable from Step 2 to Step 3 (i.e., after adding informant‐rated trait scores).

*p < 0.05, **p < 0.01, ***p < 0.001.

#### 3.3.1 Self‐report criterion variables

In Step 3 of the model for the hopelessness criterion, significant negative predictive associations were evident for both self‐reported Boldness (*β* = −0.30, *p* < 0.001) and informant‐rated Boldness (*β* = −0.19, *p* < 0.01), with positive predictive relations evident for self‐reported but not informant‐rated Meanness (*β* = 0.23, *p* < 0.001; see Table 3, top part). Adding self‐report scores to this model at Step 2 increased variance explained by 14.7% (*p* < 0.001), and adding informant ratings at Step 3 increased variance explained by 2.7% (*p* < 0.05). Substance use problems were predicted by higher levels of self‐reported Disinhibition (*β* = 0.49, *p* < 0.001) and informant‐rated Meanness (*β* = 0.17, *p* < 0.05), with the addition of self‐report scores at Step 2 and informant ratings at Step 3 accounting for 22.1% and 3.9% additional variance in substance problems, respectively (*p*s < 0.001). For self‐harm frequency, only self‐reported Disinhibition and Boldness were significant in the Step 3 model (*β*s = 0.23 and −0.12, *p*s < 0.01 and 0.05, respectively). Informant‐rated Disinhibition fell just short of significance (*β* = 0.17, *p* = 0.05). While the incremental contribution of self‐report trait scores at Step 2 was significant (Δ*R* ^2^ = 0.10, *p* < 0.001), the addition of informant‐reported traits at Step 3 was not (Δ*R* ^2^ = 0.01, *p* = 0.17). Self‐harm versatility was associated with higher levels of both self‐ and informant‐reported Disinhibition (*β* = 0.23, *p* < 0.01 and *β* = 0.18, *p* < 0.05, respectively), but again, self‐report added significantly to the variance explained (Δ*R* ^2^ = 0.07, *p* < 0.001) whereas informant report did not (Δ*R* ^2^ = 0.01, *p* = 0.14).

#### 3.3.2 Informant‐rated criterion variables

In the model for ratings of behavior in prison, both self‐ and informant‐rated Disinhibition showed negative predictive relations at Step 3 (*β* = −0.17, *p* < 0.01 and *β* = −0.37, *p* < 0.001, respectively). Self‐reported and informant‐rated Boldness, on the other hand, predicted behavior ratings in opposing directions at Step 3, with self‐reported Boldness predicting *lower* (less favorable) ratings (*β* = −0.11, *p* < 0.05) and informant‐rated Boldness predicting *higher* (more favorable) ratings (*β* = 0.13, *p* < 0.05; see Table 3, middle part). For this criterion variable, adding self‐report trait scores to the model at Step 2 and informant‐report traits at Step 3 produced a 9.0% and 11.1% increase in variance explained, respectively (*p*s < 0.001). A similar pattern was observed in the model for social‐connectivity ratings, which showed a significant increase of 7.8% in variance explained (*p* < 0.001) with the addition of informant trait scores at Step 3: Although higher levels of informant‐rated Disinhibition were associated with lower ratings of social connectivity at Step 3 (*β* = −0.24, *p* < 0.01), higher informant‐rated Boldness predicted elevated ratings of social connectivity at this step (*β* = 0.20, *p* < 0.01). By contrast, adding self‐report scores to the model at Step 2 did not significantly increase variance explained (Δ*R* ^2^ = 0.01, *p* = 0.20), and none of the self‐reported traits were predictive of social connectivity ratings at Step 3. Likewise, for reintegration prognosis, both informant‐rated Meanness (*β* = −0.23, *p* < 0.01) and Disinhibition (*β* = −0.34, *p* < 0.001) were predictive of lower ratings of expected reintegration into society, whereas informant‐rated Boldness predicted more positive expectations (*β* = 0.15, *p* < 0.01). The addition of self‐report scores at Step 2 and informant ratings at Step 3 resulted in significant increases of 8.0% and 14.6%, respectively, in variance explained (*p*s < 0.001). Of note, self‐reported Disinhibition was also predictive of lower reintegration ratings at Step 3 (*β* = −0.21, *p* < 0.01).

#### 3.3.3 Years of sentence

In the model for years of sentence (Table 3, bottom part), no additional variance was explained with the entry of self‐report trait scores at Step 2 (Δ*R* ^2^ = 0.00, *p* = 0.93), but adding informant‐rated traits at Step 3 produced a 3.4% increase in variance explained (*p* < 0.01). However, only informant‐rated Boldness evidenced significant prediction at this final step (*β* = 0.19, *p* < 0.01); neither self‐reported Boldness, nor Meanness or Disinhibition assessed either by self‐report or informant rating, showed a significant predictive association (see Table 3).

## 4 DISCUSSION

The current study sought to investigate, in a correctional sample, the extent to which brief prototype‐based informant ratings of boldness, meanness, and disinhibition covary with self‐report scores on these traits and show evidence of predictive validity in relation to self‐ and informant‐rated criterion measures—unto themselves, and over/above their self‐report counterparts.

### 4.1 Self/informant convergence

In line with study hypotheses, informant ratings of boldness and disinhibition showed significant convergence with their counterpart self‐report scores, indicating that case managers were able to recognize and effectively quantify these traits in prisoners based on brief prototypic descriptions of each; results (as discussed below) were more equivocal for meanness. As expected, however, lower self/informant convergence was observed for these traits in our study than in prior studies that have obtained informant ratings of psychopathy using detailed item‐based measures. Specifically, lower convergence was observed for boldness in the current study (*β*_B_ = 0.26) than in a study of forensic patients by Gerbrandij et al. (2019) in which informant triarchic‐rating scores were computed from personality pathology items rated by staff members (*r* = 0.45). Self/informant convergence for boldness in our study was also lower than that reported by Kelley et al. (2018) for roommate dyads who completed self‐ and other‐versions of the TriPM (*r* = 0.52). The convergence for the trait of disinhibition in our study (*β*_D_ = 0.30) was modestly higher than for boldness, and less discrepant from the figures of 0.34 and 0.36 reported by Gerbrandij et al. (2019) and Kelley et al. (2018), respectively. As mentioned previously, however, other studies used multi‐item measures for both self‐ and informant‐report, with item content consistent across report modalities. In the current study, the two assessments differed in both report type and data source (i.e., item‐based self‐report scales vs. global informant ratings), further complicating interpretation of self/informant concordance within this study and comparing our findings to those from other studies.

Although challenging to adequately compare across studies, the convergence between informant‐rated and self‐report Meanness was somewhat lower (*β*_M_ = 0.18) than that reported by Gerbrandij et al. (2019; *r* = 0.23), and appreciably lower than that reported by Kelley et al. (2018; *r* = 0.54). Moreover, in a regression model that included informant ratings for all three triarchic traits as predictors of self‐reported meanness, the beta coefficient for informant‐rated meanness fell short of significance—indicating a lack of selective self/informant convergence for this triarchic trait. This was in contrast to informant‐rated boldness and disinhibition, both of which evidenced unique predictive associations with their self‐report counterparts in corresponding regression models.

Of note, other studies have also reported lower cross‐modality convergence for assessments of meanness‐related attributes as compared to bold or disinhibited characteristics. For example, Carnovale et al. (2019) obtained self‐ and informant assessments of five broad domains of personality pathology—negative affect, disinhibition, antagonism, detachment, and psychoticism—and reported the lowest self‐other convergence for antagonism (*r* = 0.27), the domain most related to meanness (Strickland et al., 2013). Similarly, in a sample of couples with histories of partner violence who completed an inventory of psychopathy‐related traits, Iyican and colleagues (2015) found that self and spouse‐report scores diverged most strongly for traits most closely aligned with meanness (Drislane et al., 2014). In another study that specifically assessed the triarchic traits through both self‐report and informant ratings, Gerbrandij et al. (2019) found lower cross‐modality agreement for meanness (*r* = 0.23) than for either Boldness (*r* = 0.45) or Disinhibition (*r* = 0.34). These authors postulated that informant ratings were more influenced by observable, behavioral‐deviancy features of meanness (e.g., aggression, insolence) whereas self‐report scores included greater representation of internal, affective‐deficiency features (e.g., lack of empathy, guiltlessness).

The same interpretation can be applied to the findings of the current study, particularly given our use of global, prototype‐based informant ratings. The Meanness scale of the self‐report based TriPM includes a substantial portion of items that assess emotional sensitivity and empathic concern (e.g., “I don't have much sympathy for people”), along with items pertaining to more observable indicators of callousness (e.g., “I enjoy pushing people around sometimes”). Case managers, on the other hand, especially those less familiar with the participant to be rated, would be expected to base their global meanness ratings predominantly on observable behaviors indicative of antagonism and callous‐disregard (e.g., arrogance, defiance of authority) that overlap more with disinhibition. In fact, informant ratings exhibited stronger inter‐correlations compared to self‐report scores (i.e., *r* = 0.31 vs. 0.16 for Boldness and Meanness; *r* = 0.26 vs. −0.02 for Boldness and Disinhibition; *r* = 0.65 vs. 0.55 for Meanness and Disinhibition), and these augmented associations may be a result of informants' reliance on overt behavior in assigning their ratings, compared to the prisoners who based their self‐report on internal experience as well. This discrepancy may be especially relevant to the meanness domain, which has fewer unique interpersonal and behavioral indicators compared to boldness and disinhibition.

Other studies have attributed discrepancies between self‐report and other‐ratings to differences in overall psychopathy levels, citing concerns about the validity of self‐report in psychopathic individuals. For example, Carnovale and colleagues found that self‐informant discrepancies increased with escalating severity of general personality pathology (Carnovale et al., 2019). Likewise, in a sample of incarcerated women enrolled in a substance use treatment program, Jackson and Richards (2007) reported greater self/other discordance among participants exhibiting greater affective features of psychopathy (i.e., callousness, shallow affect). Similarly, Kelley et al. (2018) found that higher total TriPM scores were associated with greater divergence between self‐report and informant scores. Future research should continue to consider the potential moderating effect of overall pathology levels on convergence between informant‐rating and self‐report assessments.

### 4.2 Predictive validity of informant ratings

As hypothesized, informant ratings also demonstrated predictive validity in relation to criterion measures, largely in directions consistent with previously reported associations for self‐report triarchic scores. Our finding that higher ratings of boldness were related to lower levels of hopelessness aligns with results for TriPM Boldness reported by Brislin et al. (2019) as well as with findings of negative relations between self‐reported boldness and measures of internalizing psychopathology in other studies (Latzman et al., 2019, 2020). In contrast, higher ratings of boldness were associated with more favorable ratings of behavior in prison, better social connectedness, and more positive expectations for reintegration into society. Despite the multitude of negative outcomes associated with psychopathy, such as risk for criminal recidivism or perpetration of violence (Douglas et al., 2018), theoretical accounts of psychopathy have underscored the potential adaptive value of boldness‐related features, including interpersonal charm, social poise, and stress immunity (Benning et al., 2005; Patrick et al., 2009). These features have famously been characterized as a “mask of sanity” that functions to conceal underlying affective‐interpersonal and behavioral deficits, through a guise of affability and positive adjustment (Cleckley, 1976; Patrick, 2018). As such, it seems likely that informant ratings of boldness in the current study were influenced to an important degree by behavioral indicants of positive adjustment such as charm, social confidence, and emotional stability—features that have long been considered important for differentiating psychopathy from other impulsive‐externalizing conditions (Crego & Widiger, 2016; Lilienfeld et al., 2016; Venables et al., 2014). Of note, the years of sentence criterion variable was related positively and uniquely to boldness. This lends further support to the idea of boldness as an effective “mask of sanity:” Individuals rated higher in boldness, despite having longer sentences indicative of more serious crimes, were perceived as better behaved, more socially interconnected, and more likely to reintegrate successfully into society (see also results for boldness in the next subsection on Section 4.3).

Nevertheless, not all psychopathy researchers agree that boldness is a core, defining feature of psychopathy, and spirited debate has surrounded this topic for some time now (e.g., Berg et al., 2017; Crego & Widiger, 2015; Lilienfeld et al., 2016; Sleep et al., 2019). Some scholars contend that the adaptive correlates of boldness, such as lower hopelessness and more favorable staff ratings in the current study, are inconsistent with the clinical nature of psychopathy and its established associations with impulsive‐antisocial behaviors (Miller & Lynam, 2012; Sleep et al., 2019). Although boldness and disinhibition (e.g., externalizing proneness) are conceptualized as orthogonal dispositions (Lilienfeld et al., 2019; Patrick et al., 2009), and emerge as such in latent space (e.g., Drislane & Patrick, 2017), research increasingly demonstrates that these traits are not incompatible. In fact, a growing body of work has demonstrated interactive effects of the two (e.g., Latzman et al., 2019, 2020), consistent with the idea that the combination of high boldness and high disinhibition is typified by a low internalizing, extra‐high externalizing profile akin to Cleckley's (1976) “mask of sanity” concept (Patrick, 2018, in press). At the very least, the present study demonstrates that self‐ and informant‐ratings of boldness meaningfully predict clinical and social criterion variables in a forensic context, underscoring the value of considering this trait alongside meanness and disinhibition.

Informant‐rated disinhibition also exhibited expected patterns of relations with criterion variables. As reported by Brislin et al. (2019) for TriPM Disinhibition, and consistent with findings from many other studies (Iacono et al., 1999; Joyner et al., 2019, 2020), informant‐rated disinhibition was associated robustly with substance use problems in the current sample—both when examined in an individual‐trait regression model and when examined concurrently with informant‐rated boldness and meanness in a joint model. Informant‐rated disinhibition also showed positive, preferential relations with both frequency and diversity of self‐harm behavior, mirroring results for TriPM Disinhibition in the current sample (Brislin et al., 2019). Other work has shown that self‐reported disinhibition and facets of impulsivity related to it are predictive of suicidal behavior (Gottfried et al., 2019; Tylicki et al., 2019) as well as non‐suicidal self‐injury (Glenn & Klonsky, 2010). Higher informant ratings of disinhibition in the current study were also related to less favorable perceptions of behavior in prison, lower social connectedness, and poorer reintegration prognosis. The finding that case managers had poorer perceptions of and expectations for behavior in regard to high‐disinhibited individuals fits with the concept of disinhibition as general externalizing proneness (Patrick et al., 2009), and with empirical work linking this trait to a broad range of norm‐violating behaviors (Krueger et al., 2007; Nelson & Foell, 2018).

Compared to boldness and disinhibition, informant ratings of meanness exhibited fewer predictive relations with criterion measures. Although meanness ratings on their own were negatively associated with ratings of behavior in prison and social connectivity, these associations were nonsignificant in joint regression models that included all three informant‐rated traits as predictors. As noted in the preceding paragraph, coefficients for disinhibition remained significant in these regression models, indicating that the individual‐trait beta coefficients for meanness were attributable to variance shared with disinhibition. However, in joint regression models, higher ratings of meanness did significantly predict more substance problems and less favorable ratings of reintegration prognosis—indicating a unique contribution to prediction of these criteria over/above disinhibition. Meanness is conceptualized as a reflection of low affiliative capacity (Palumbo et al., 2020; Viding & McCrory, 2019) and encompasses features like social detachment, rebelliousness, and exploitativeness (Drislane et al., 2014; Patrick et al., 2009), all of which may factor negatively into problematic substance use and informant expectations for successful reentry into society.

### 4.3 Incremental validity of informant ratings

Finally, we used hierarchical regression analyses to evaluate whether prototype‐based informant ratings of boldness, meanness, and disinhibition provided unique predictive information beyond that afforded by item‐based scale self‐report measures of these traits. Analyses revealed that, for all informant‐rated criterion variables and two of the four self‐report criteria, the addition of informant ratings resulted in a significant increase in variance explained.

Among the self‐report criteria, informant ratings evidenced incremental validity in the prediction of hopelessness and substance problems, but not self‐harm frequency or versatility. Interestingly, in the model with all self‐report and informant‐rated traits entered as predictors of substance problems at Step 3, the only significant beta coefficients were for self‐report disinhibition and informant‐rated meanness, with no unique contribution of informant‐rated disinhibition. This contrasts with the regression analysis for informant‐rated traits alone, in which disinhibition emerged as the strongest predictor of substance problems. These comparative results indicate that informant‐rated disinhibition contributed to prediction of substance problems mostly as a function of variance shared with self‐reported disinhibition. By contrast, the significant contribution of informant‐rated meanness at Step 3 of the hierarchical model reflected variance separate from self‐report meanness, which was entered at Step 2. Invoking our earlier interpretation regarding weak self/informant convergence for meanness, a plausible explanation is that overt‐behavioral indicants represented more in informant‐ratings of this trait than in self‐report (e.g., arrogance or defiance) may account for its unique predictive relation with substance problems.

Notably, all informant‐rated criterion variables benefited from the addition of informant ratings at Step 3 of the hierarchical regression model. Interestingly, in predicting ratings of behavior in prison, the beta coefficients observed for self‐ and informant‐rated boldness were in opposing directions, such that higher informant‐perceived boldness was associated with significantly higher (more favorable) behavior ratings, whereas higher self‐perceived boldness was associated with lower (less favorable) behavior ratings. Paralleling our interpretation for meanness, this divergence may be a function of differing features entering into informant‐rated boldness as compared to self‐reported boldness. Like meanness, boldness encompasses features pertaining to internal experience (e.g., immunity to stressors, tolerance of uncertainty) along with behaviorally observable features (e.g., charm, social assertiveness). Consistent with the idea of boldness as a “mask of sanity,” the overt features of boldness could be expected to contribute more uniformly to impressions of positive adjustment on the part of observers.

Importantly, in these hierarchical regression models, the validity coefficients for informant‐rated traits were generally stronger for informant‐rating criterion variables (range of |*β*| = 0.13–0.37) than for self‐report criteria (range of |*β*| = 0.17–0.19). The reverse was true for validity coefficients associated with self‐report trait scores, which were generally stronger for self‐report criterion variables (range of |*β*| = 0.12–0.49) than for informant‐rating criteria (range of |*β*| = 0.11–0.21). Likewise, the incremental contribution of informant‐rated traits was larger for predicting informant‐rating criteria (Δ*R* ^2^s = 0.08–0.15) than for predicting self‐report criteria (Δ*R* ^2^s for hopelessness and substance use problems = 0.03 and 0.04, respectively) or years of sentence (Δ*R* ^2^ = 0.03). Again, the reverse was true for self‐report, in that the incremental contribution of self‐reported trait scores was larger for predicting self‐report criteria (Δ*R* ^2^s = 0.07–0.22) than for predicting informant‐rated criteria (Δ*R* ^2^s for behavior and reintegration ratings = 0.09 and 0.08, respectively), and self‐report did not significantly add to the variance explained for years of sentence. These differential effects are theoretically coherent given the well‐documented impact of method variance on validity coefficients in prior research of this kind (e.g., Blonigen et al., 2010; Gerbrandij et al., 2019; Iyican et al., 2015).

### 4.4 Clinical and practical implications

The current findings have promising clinical and practical implications for psychopathy assessment in correctional settings. First, our findings demonstrate that simple, prototype‐based informant ratings of the triarchic traits can feasibly be obtained in a forensic context, where personnel and resources are typically limited. Modest convergence between self‐report and informant ratings suggests that global informant ratings may be less useful for corroboration of or as a substitute for self‐report measures. Rather, given the incremental contributions of informant scores over and above self‐report in predicting a variety of clinical outcomes, these simplified informant ratings may be most useful as a supplement to self‐report triarchic scores. Moreover, self/other agreement was higher for boldness and disinhibition compared to meanness, which also evidenced more variable predictive validity. It is possible that the characteristics used by case managers to rate meanness in the current study differed from those indexed by the TriPM meanness scale, and that convergence between the two modalities could be enhanced by training raters to rely on a broader range of cues. However, doing so would be more complex and burdensome, and there may be elements of meanness that are inherently less accessible to observers, limiting the potential improvement in self/other convergence. It is also possible that the highly‐controlled environment of the prison may operate to limit or moderate expressions of meanness in ways that affect the validity of staff‐informant ratings. For example, given the penalties applied to hostile, aggressive, and exploitative behavior in correctional settings, meanness may tend to be expressed more as aloofness and lack of participation in available programs and activities/events.

In general, introducing more complex rating procedures, such as increasing the number of raters or scale items, may bolster self/other correspondence by reducing measurement error or providing more comprehensive content coverage (Schmidt & Hunter, 1996). These advantages may be especially relevant for assessing affective‐interpersonal traits like boldness or meanness, which may manifest internally via subjective experience (e.g., perceived superiority over others for boldness, or apathy toward others for meanness) or externally via observable behavior (e.g., interpersonal charm for boldness, or proactive aggression for meanness). On the other hand, the low time commitment and easy implementation of single‐item, single‐rater informant scores, as employed in the current study, offer significant practical advantages in low‐resourced settings such as prisons, especially considering the complementary information they provide alongside self‐report. Overall, the availability of a low burden, informant‐based psychopathy assessment may provide a pragmatic and cost‐efficient means to improve the validity of prisoner evaluations that frequently factor into critical forensic and legal decisions (e.g., institutional assignment, parole eligibility, participation in programs).

### 4.5 Limitations and future directions

Although the present study had several strengths (e.g., large, mixed‐sex sample of incarcerated individuals across multiple prisons), several limitations must be noted as well. First, limited data were available for characterizing the study informants, in terms of their familiarity with each rated participant, their degree of experience serving in a case manager role, and other factors that could have influenced ratings. As a result, we were unable to evaluate these factors as potential moderators of self/informant convergence and the predictive validity of informant ratings. Future studies of this kind should explicitly assess how the extent of contact between prisoners and informant prison staff affects agreement across report modalities. Along similar lines, detailed demographic data were not available for the prisoners, preventing a more comprehensive look at potential moderators of the relationships observed in this study (apart from biological sex, as examined by Sica et al., 2021).

Second, it was not possible to collect ratings from multiple informants per participant, thus precluding evaluation of the interrater reliability of the prototype‐based rating procedure. In particular, given the single‐item nature of these ratings, it seems likely that measurement error contributed to the modest cross‐modality associations we observed. However, as discussed in the preceding section, the practical advantages of this simplified rating approach may outweigh the costs of obtaining more than one set of ratings per case. Even so, future work should explore the psychometric benefits versus time‐expenditure cost of collecting ratings from different informants or providing raters with training in the triarchic model of psychopathy. In addition, it will be important in future research to evaluate the benefits versus costs of a more granular informant‐rating approach (e.g., rating the degree to which different items pertaining to each triarchic trait apply to a target individual). Of note, a recent study by Sica et al. (2020) that employed shortened TriPM scales (10‐11 items for each trait) reported acceptable internal consistency and good correspondence with the full‐length scales. A fruitful avenue for future research would be to compare the administration time of an informant‐rating version of this shortened TriPM with that of the prototype rating approach used in the current study, evaluating the reliability and criterion‐related validity of the two approaches.

Finally, similar to other studies of self and informant‐rated psychopathic traits, the current findings are limited to a research setting in which neither the incarcerated individuals nor the informant raters faced consequences for inaccurate reporting. Thus, while incentive to provide false report in the current study was low, it is unclear whether findings would generalize to real‐world settings where the incentive to misrepresent is higher (e.g., evaluations for sentencing or parole eligibility).

## 5 CONCLUSION

Notwithstanding these limitations, the current study provides support for the use of a simple global‐rating approach to collecting collateral data for boldness, meanness, and disinhibition as described by the triarchic model of psychopathy (Patrick et al., 2009). While the overlap between self‐ and informant‐rated triarchic traits is limited, informant ratings exhibit significant predictive utility in relation to a variety of criterion measures, and incrementally contribute to predicting the majority of these outcomes over and above self‐report. As such, our findings suggest that a simplified, low‐burden procedure for rating the triarchic traits can serve as a useful supplement to self‐report triarchic scores, and help mitigate concerns about the use of self‐report assessment measures in forensic and legal settings.

## CONFLICT OF INTEREST

The authors declare no conflict of interest.

## Supporting information

Supplementary Material

## ENDNOTE

1 Supplemental Table A shows associations between informant‐rated triarchic traits and criterion variables for male and female prisoner subgroups; it can be seen that associations were similar in most cases. This accords with the finding of similar relations for self‐report (TriPM) trait scores with study criteria across these subgroups, as reported by Sica et al. (2021).
