# Supplementary material for: Evaluating the validity of brief prototype‐based informant ratings of triarchic psychopathy traits in prisoners
Source: Behav Sci Law. 2021 Oct 17;39(5):641–62. doi: 10.1002/bsl.2542 (PMC9297945; doi:10.1002/bsl.2542)
Supplement: Supplementary file 1 — Supplementary Material [file BSL-39-641-s001.docx]

**Evaluating the validity of brief prototype-based informant ratings of triarchic psychopathy traits in prisoners**

***Supplemental Material***

*Contents*

**SUPPLEMENTAL METHOD A.** Prototypic descriptions of boldness, meanness, and disinhibition (English)

p. 2

**SUPPLEMENTAL METHOD B.** Prototypic descriptions of boldness, meanness, and disinhibition (Italian)

p. 3

**SUPPLEMENTAL TABLE A.** Correlations between informant-rated triarchic traits and criterion variables for male and female subgroups

p. 4

**SUPPLEMENTAL METHOD A.** Prototypic descriptions of boldness, meanness, and disinhibition (English)

***Boldness:*** This person can be defined as essentially socially dominant, confident, immune from stress, a lover of adventure, not at all concerned by novel and uncertain situations, courageous and daring. He/she is characterized by staying calm and focused even in threatening situations. With others he/she can appear very confident and play the role of leader. Extroverted and comfortable in social situations, he/she possesses excellent skills of dialogue and persuasion. Others are often attracted to this type of person. Such individuals can also be described as optimistic, having great self-confidence, and unlikely to be discouraged in adverse situations. They do not worry about possible future problems, and even in the face of negative events or bad luck, they do become disheartened and are able to recover quickly.

***Meanness:*** These individuals are characterized by insensitivity and a lack of interest in other people’s well-being or emotions. They consider themselves superior to others, and they are not interested in close relationships or emotional attachments. They tend to exploit others; they are aggressive and in order to get what they want and can behave in cruel and destructive ways. They are arrogant, they mock others, and they tend to challenge and provoke others, and be very competitive. They have little respect for authority and generally have no form of deep relationship with anyone. Uninterested in the pain of others, they try to achieve what they want by harming and exploiting others and making them suffer. They take pleasure from the suffering of others, cruel to people and animals. They show no affection, they are cynical and vindictive and often consider emotions as a sign of weakness. They do not cooperate with others or collaborate: their philosophy is summed up in the idea that "the strongest wins" and that the world is a place where "dog eats dog." Such individuals find excitement in activities that involve risk or danger.

***Disinhibition:*** This person is characterized by being impulsive, unable to reflect on or inhibit his/her own behaviors, lacking in planning skills, and unmindful of potential consequences for his/her own behaviors. Having unstable mood and limited ability to tolerate frustration, he/she gets bored easily and tends to react aggressively to frustrations and provocations. Such individuals are irresponsible, and often do not fulfill work duties or family commitments. Being interested in the immediate gratification of his/her needs, he/she reacts aggressively if those needs are not met quickly. He/she can also be described as inconsistent, untrustworthy, and distrustful. He/she tends to consistently violated rules and laws. Unreliable and impatient, his/her life is often chaotic and disorganized. He/she often lacks self-control and makes important decisions without considering the consequences. Unable to regulate his/her mood, he/she may resort to drugs, alcohol, or medication to feel better. Such individuals tend to blame others for the negative consequences that arise from their behavior.

**SUPPLEMENTAL METHOD B**. Prototypic descriptions of boldness, meanness, and disinhibition (Italian)

***Audacia:*** Questa persona può essere definita come sostanzialmente dominante, sicura di sé, apparentemente immune dallo stress, amante delle avventure e delle emozioni forti, per nulla preoccupata dalle novità e dalle situazioni incerte, coraggiosa ed intrepida. Si caratterizza per rimanere calma e concentrata anche nelle situazioni minacciose. Con gli altri può apparire molto sicura e ricoprire il ruolo di leader. Estroversa e a proprio agio nelle situazioni sociali, possiede ottime abilità di dialogo e persuasione. Gli altri sono spesso attratti da questo tipo di persona. Possono anche essere descritte come persone ottimiste, con grande fiducia in se stesse, che difficilmente si scoraggiano nelle situazioni avverse. Non si preoccupano di possibili problemi futuri e anche in caso di eventi negativi o sfortuna, non li lasciano abbattere e si riprendono rapidamente.

***Disinibizione:*** Questa persona si caratterizza per essere impulsiva, incapace di riflettere e di inibire i propri comportamenti, mancante di abilità di pianificazione, apparentemente noncurante delle conseguenze a lungo termine dei propri comportamenti. Di umore instabile, con scarsa capacità di tollerare la frustrazione, si annoia facilmente e tende a reagire in modo aggressivo alle frustrazioni e alle provocazioni. Irresponsabile, spesso non assolve i propri doveri lavorativi né quelli familiari. Interessata alla immediata gratificazione dei propri bisogni, reagisce in modo aggressivo se le sue esigenze non sono soddisfatte rapidamente. Può anche essere descritta come incostante, sospettosa e diffidente. Tende all’antisocialità, alla sistematica trasgressione delle regole e delle leggi. Inaffidabile e impaziente, spesso la sua vita è caotica e disorganizzata. Prende decisioni importanti senza valutarne le conseguenze e manca spesso di auto-controllo. Incapace di regolare il proprio umore, può ricorrere a droghe, alcol o farmaci per sentirsi meglio. Tende a colpevolizzare gli altri per le conseguenze negative che il proprio comportamento spesso genera.

***Cattiveria:*** Queste persone si caratterizzano per insensibilità e nessun interesse per il benessere o le emozioni altrui. Si considerano superiori agli altri, non sono interessate alle relazioni con gli altri né all’attaccamento emotivo. Tendono a sfruttare gli altri, sono aggressive e al fine di ottenere ciò che vogliono possono comportarsi in modo crudele e distruttivo. Sono arroganti, deridono gli altri, tendono a sfidare e a provocare gli altri e ad essere molto competitive. Non hanno nessun rispetto per l’autorità e non hanno in genere alcuna forma di relazione profonda con qualcuno. Disinteressate al dolore altrui, cercano di ottenere ciò che vogliono danneggiando e sfruttando gli altri e facendoli soffrire. Traggono piacere dalla sofferenza altrui, crudeli con persone e animali. Non mostrano affetto, sono ciniche e molto vendicative e spesso considerano le emozioni come un segno di debolezza. Non cooperano con gli altri né collaborano: la loro filosofia è riassunta nell’idea che “il più forte vince” e che il mondo è un posto dove “cane mangia cane.” Amano le attività anche molto rischiose e pericolose.

**SUPPLEMENTAL TABLE A.** Associations between informant-rated triarchic traits and criterion measures for male and female participant subgroups

| Criterion Measure | | Informant rating | | | | | |
| --- | --- | --- | --- | --- | --- | --- | --- |
|  |  | Males (n = 246) | | | Females (n = 76) | | |
|  |  | β_B_ | β_M_ | β_D_ | β_B_ | β_M_ | β_D_ |
| Self-report criteria | |  |  |  |  |  |  |
|  | TriPM Boldness (B) | .23^**^ | .05^M^ | .04^D^ | .33^**^ | .31^*M^ | .40^**D^ |
|  | TriPM Meanness (M) | -.03 | .15 | .09 | .17 | .26 | .34* |
|  | TriPM Disinhibition (D) | .04 | .21^**^ | .29^***^ | -.02 | .20 | .33* |
|  | Hopelessness | -.28^***^ | .04 | -.06 | -.16 | .06 | .15 |
|  | Self-harm frequency | -.08 | .05^M^ | .12^D^ | .09 | .32^*M^ | .40^**D^ |
|  | Self-harm versatility | -.08 | .05 | .13 | .00 | .21 | .31^*^ |
|  | Substance Use Problems | .14^*^ | .34^***^ | .31^***^ | .19 | .36^**^ | .46^***^ |
| Informant-rated criteria | |  |  |  |  |  |  |
|  | Behavior in prison | .00 | -.35^***M^ | -.43^***D^ | .00 | -.51^***M^ | -.62^***D^ |
|  | Social connectivity | .17^*^ | -.23^**^ | -.23^**^ | -.06 | -.36^**^ | -.44^**^ |
|  | Reintegration prognosis | -.01 | -.45^***^ | -.52^***^ | -.06 | -.52^***^ | -.53^***^ |
| Years of sentence | | .20^**^ | .12 | .03 | .13 | .10 | .08 |

Note: TriPM = Triarchic Psychopathy Measure. Beta coefficients (βs) are from individual-trait regression models including scores for one of the informant-rated triarchic traits, along with dummy-coded variables representing different raters, as predictors. Paired superscripts within a row denote βs for a given trait rating (^B^=Boldness, ^M^=Meanness, ^D^=Disinhibition) that differed significantly for males versus females **(***p* < .05).

* p < .05, ** p < .01, *** p < .001
